# Substance Use Outcomes Among Sexual and Gender Minority Individuals Living with HIV Following Residential Substance Use Treatment in Washington, DC

**DOI:** 10.1080/07347324.2023.2241419

**Published:** 2023-08-08

**Authors:** Jennifer M. Belus, Hannah Tralka, Emily N. Satinsky, C.J. Seitz-Brown, Stacey B. Daughters, Jessica F. Magidson

**Affiliations:** a Department of Clinical Research, Division of Clinical Epidemiology, University Hospital Basel, Basel, Switzerland; b University of Basel, Basel, Switzerland; c Department of Psychology, University of Maryland, College Park, MD, USA; d Department of Behavioural and Community Health, University of Maryland, College Park, MD, USA; e Department of Psychology, University of Southern California, Los Angeles, California, USA; f Department of Psychology and Neuroscience, University of North Carolina at Chapel Hill, Chapel Hill, NC, USA; g Center for Substance Use, Addiction & Health Research (CESAR), University of Maryland, College Park, MD, USA

**Keywords:** sexual minority, gender minority, HIV, substance use, minority stress

## Abstract

This study explored how sexual or gender minority (SGM) status influenced substance use (SU) treatment outcomes in a predominantly African American and unemployed sample of people with HIV. *N* = 60 participants were enrolled in an abstinence-focused inpatient SU treatment center, followed by outpatient treatment sessions. At 12-months follow-up, the survival rate (i.e. those who did not reuse substances) was 37.6% (non-SGM group) vs. 4.8% (SGM group). The impact of SGM status on reuse was .54 log odds, *p* = .11, which translates to a 71.8% increase in the hazard of reusing substances for SGM vs. non-SGM individuals. For both groups, frequency of reuse remained stable and problems associated with SU decreased over time. Results suggest a potentially clinically relevant finding that SGM individuals have possible heigh-tened risk of SU after a mixed inpatient-outpatient program. ClinicalTrials.gov trial registration number: NCT01351454.

Compared to cisgender, heterosexual individuals, sexual and gender minority (SGM) individuals*^1^ are disproportionately affected by problematic substance use (SU). SGM individuals tend to start drinking earlier (Flentje et al., 2015; Green & Feinstein, 2012; Greenwood & Gruskin, 2007; Hughes & Eliason, 2002; McCabe et al., 2013), demonstrate a higher frequency of SU (Cochran & Cauce, 2006; Flentje et al., 2015), and have approximately twice the odds of having a lifetime SU disorder (Kerridge et al., 2017). Since SGM individuals are at an increased risk of SU disorders due to the minority stressors they face in a discriminatory society, it is important to ensure that SU treatment is responsive to their unique needs.

Furthermore, SGM individuals can have multiply minoritized statuses, such as having an HIV-positive serostatus, a low socioeconomic status, or being from a minoritized racial or ethnic group. Multiply minoritized SGM individuals are at a higher risk for problematic SU compared to those without multiply minoritized statuses due to the multiple, intersecting forms of discrimination that they face (Baptiste-Roberts & Hossain, 2018; Bing et al., 2001; Díaz et al., 2001; English et al., 2018; McCabe et al., 2010; McKirnan et al., 2001; Turner & Wallace, 2003; Vilsaint et al., 2019). One study in an SGM sample found that nearly 50% of individuals who reported discrimination on the basis of multiply minoritized statuses met criteria for an SU disorder in the past year, compared to less than 20% of individuals who reported no discrimination on the basis of having multiple minoritized statuses (McCabe et al., 2010).

This intersectionality is important to consider, as different forms of stigma against people with multiply minoritized statuses are multiplicative and interdependent (Bowleg, 2008; Hill Collins & Bilge, 2016). SGM individuals who also have minoritized racial/ethnic identities can experience stress from both homophobia and racism, putting them at a higher risk for adverse health conditions (Balsam et al., 2011). A longitudinal study of sexually active racial minority SGM men in New York City found that the interaction of racial discrimination and “gay rejection sensitivity,” the extent to which someone expected and perceived rejection based on their gay identity, predicted higher levels of heavy drinking (English et al., 2018).

Although SGM individuals exhibit higher treatment seeking behaviors for SU than non-SGM individuals (Drabble et al., 2005; Lea et al., 2013; Li, 2018; McCabe et al., 2013), there is limited research on how SGM status affects SU treatment outcomes. The available evidence is mixed, with some studies identifying worse treatment outcomes among SGM individuals (Senreich, 2007, 2009, 2011), some showing mixed results (Cochran, 2004), and others showing no differences (Senreich, 2012). Research is also lacking on examining SU treatment outcomes among individuals who have multiply minoritized statuses. For example, the majority of prior research on SGM treatment outcomes has used 50% or more White samples (cf. Senreich, 2012).

To better understand how SGM individuals fare in SU treatment programs, this study conducted a preliminary analysis of how SGM status influenced time to substance reuse, frequency of SU, and problems associated with SU in a multiply minoritized sample, comprised largely of low-income, African American individuals, all of whom were living with HIV, who participated in a mixed inpatient-outpatient treatment program. We hypothesized that, compared to cisgender, heterosexual individuals, SGM individuals would have shorter time to substance reuse, greater frequency of SU, and greater problems associated with SU following treatment (Bruce et al., 2008; Cochran, 2004; Díaz et al., 2001; English et al., 2018).

## Materials and methods

### Participants and procedures

Data for this study were taken from a randomized clinical trial that compared a behavioral activation intervention to supportive counseling for SU. Detailed methods are described elsewhere (Magidson et al., 2022). Participants were recruited from an abstinence-focused, residential inpatient treatment center in Washington, D.C. Once discharged from the inpatient setting, which is court-mandated for ~70% of clients, participants completed additional outpatient sessions. Most treatment stays are 30 days, but court-mandated contracts may be up to 90 days.

For inclusion in the trial, inpatient clients needed to be between 18–65-years-old and living with HIV. HIV-positive status was assessed via self-report and clinic-verified records. Individuals were excluded from the study if they endorsed active, untreated psychotic symptoms; were unable to provide voluntary, informed consent; or had less than a third-grade English reading level. Eligible participants (*N* = 61) completed a baseline assessment and were randomized (1:1) to receive a 16-session intervention focused on SU and medication adherence. Participants either received (a) “Act Healthy” an intervention that combines behavioral activation for substance use plus a problem-solving focused medication adherence program called Life-Steps (Daughters et al., 2010), or (b) supportive counseling plus Life-StepsMagidson et al., 2011. Study interventionists were clinical psychology doctoral students. For the current investigation, one participant did not self-report on gender identity and was excluded from analysis, resulting in a final study sample of *N* = 60.

All participants received the residential inpatient center’s standard treatment components, including daily Alcoholics Anonymous and Narcotics Anonymous meetings, and group sessions focused on anger management, spirituality, relapse prevention, and other life skills. Participants provided informed consent prior to participating in the study, and procedures were approved by the University of Maryland Institutional Review Board.

### Measures

#### Demographics

Participants provided their age, sex, gender identity, sexual orientation, race, employment status, prior residential treatment episodes, and years since HIV diagnosis.

#### SGM status

Participants were prompted by the statement, “I describe my sexual orientation as …,” and could select from three options: heterosexual, bisexual, or homosexual*^2^ . They were also asked, “Do you identify as transgender?” (yes/no). Given the small sample sizes, participants who self-identified as gay, lesbian, bisexual, and/or transgender were categorized as SGM.

#### Any SU

SU was assessed dichotomously using combined data from urinalysis and the Timeline Follow Back (TLFB) (Sobell & Sobell, 1992). A biological indicator of SU was measured using the five-panel Integrated E-Z Split Key Cup urine screen, which tested for cocaine, amphetamines, phencyclidine (PCP), tetrahydrocannabinol (THC), and opiates. TLFB is a calendar method used to aid memory and improve self-report of SU. TLFB assessment covered the time since the last assessment. For each day, participants reported whether and how much of any substance was used. SU was coded as “yes” if participants reported any SU on the TLFB or had a positive urinalysis result. Data were collected at the following timepoints: residential treatment discharge (4 weeks), conclusion of the outpatient sessions (12 weeks; post-treatment), and 1-, 3-, 6-, and 12-months post-treatment.

#### SU frequency

Frequency of SU was examined to provide a nuanced understanding of how SU changed over time; it was determined based on TLFB data. The number of days used was divided by total days in the assessment period. TLFB data were also collected at the weekly outpatient visits, in addition to the major visits described above.

#### Problems associated with SU

The Short Inventory of Problems – Alcohol and Drugs (SIP-AD) is a 15-item self-report measure that assesses five domains of substance-related problems: impairment to physical health, interpersonal functioning, intrapersonal or emotional functioning, impulse control-related consequences, and social responsibility (Blanchard et al., 2003). Each domain includes three items rated on a four-point Likert scale ranging from *never* to *daily or almost daily*. Total scores range from 0 to 45, with higher scores indicating more problems. Data were collected at baseline, post-treatment, and 1-, 3-, 6-, and 12-months post-treatment.

#### HIV-related health

CD4 count and viral load were extracted from medical records and used as an indicator of HIV-related health.

### Data analytic plan

Descriptive statistics were calculated overall and by SGM status. We used a discrete time survival analysis to compare time to reuse between SGM and non-SGM individuals (Allison, 2010). Survival analysis allowed us to examine the probability of abstaining from SU at each time point (i.e., the survival function) as well as the risk of using substances at each time point given that an individual had not previously reused (i.e., the hazard function). A discrete time survival analysis was selected because we could not identify the exact timing of SU. We followed guidance by Allison (Allison, 2010) to use the complementary log–log model, which assumes underlying continuous-time data measured at discrete intervals. We followed Singer and Willett’s recommendations to use graphical depictions of the survival and hazard functions (J. D. Singer & Willett, 2003). SGM status was the primary predictor of interest. Time was dummy coded, and length of the assessment period and its squared term (to control for nonlinearity) were included as covariates.*^3^ Coefficients in the model can be exponentiated*^4^ (Hughes & Eliason, 2002) and interpreted as a hazard ratio. This is analogous to a proportional hazard model (Allison, 2010), which is also the standardized effect size for these models.

To estimate differences in frequency of SU and problems associated with SU over time between SGM and non-SGM individuals, we used multilevel modeling to account for repeated measurements (Raudenbush & Bryk, 2002). We used an intent-to-treat framework (Chakraborty, 2009), which included all available data in the analyses. To work with the limitation of missing data, we followed Mallinckrodt and Lipkovich’s recommendations to treat missing data as missing at random, conducting sensitivity analyses to assess the robustness of this assumption (Mallinckrodt & Lipkovich, 2016). Frequency of SU was modeled as a count variable (accounting for the assessment period length) and we used PROC GLIMMIX with Laplace estimation, which approximates maximum likelihood and produces less biased estimates in small samples. SIP-AD was modeled as a continuous variable using PROC MIXED with the restricted information maximum likelihood method. We used visual representations of the data and likelihood ratio tests to determine the appropriate shape of the trajectory to be modeled. All analyses were first conducted with SGM status as the only predictor and then, in a second model, controlling for any observed baseline differences between groups. Due to minimal differences observed in SU outcomes by treatment group (Magidson et al., 2022), we did not control for intervention received in the model. All analyses were run using SAS (2018) version 9.4.

## Results

### Demographics

Across the entire sample, the mean age was 45.38 (*SD* = 7.81), 53.3% were female (*n* = 32), 96.7% (*n* = 58) identified as African American, and 35% (*n* = 21) identified as SGM (*n* = 4 identified as gender minorities and *n* = 20 identified as sexual minorities, not mutually exclusive). The SGM and non-SGM groups differed in age and prior residential treatment episodes, with the SGM participants being younger (42.4 years vs. 47.0 years) and having more prior treatment episodes (4.7 vs. 2.7), see Table 1. Analyses controlled for these covariates.

### Time to substance reuse

**Figure 1.**
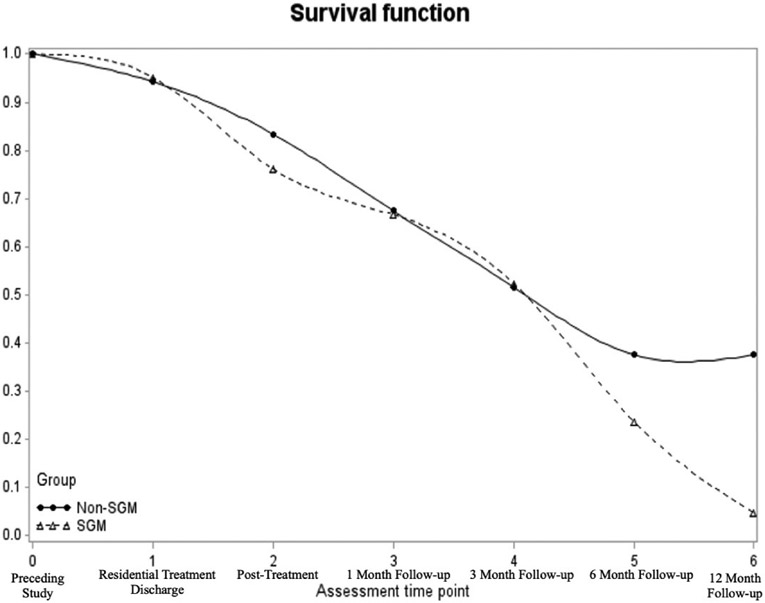
Survival function (i.e., those not using substances) by SGM status from residential discharge to 12-month follow-up.

**Figure 2.**
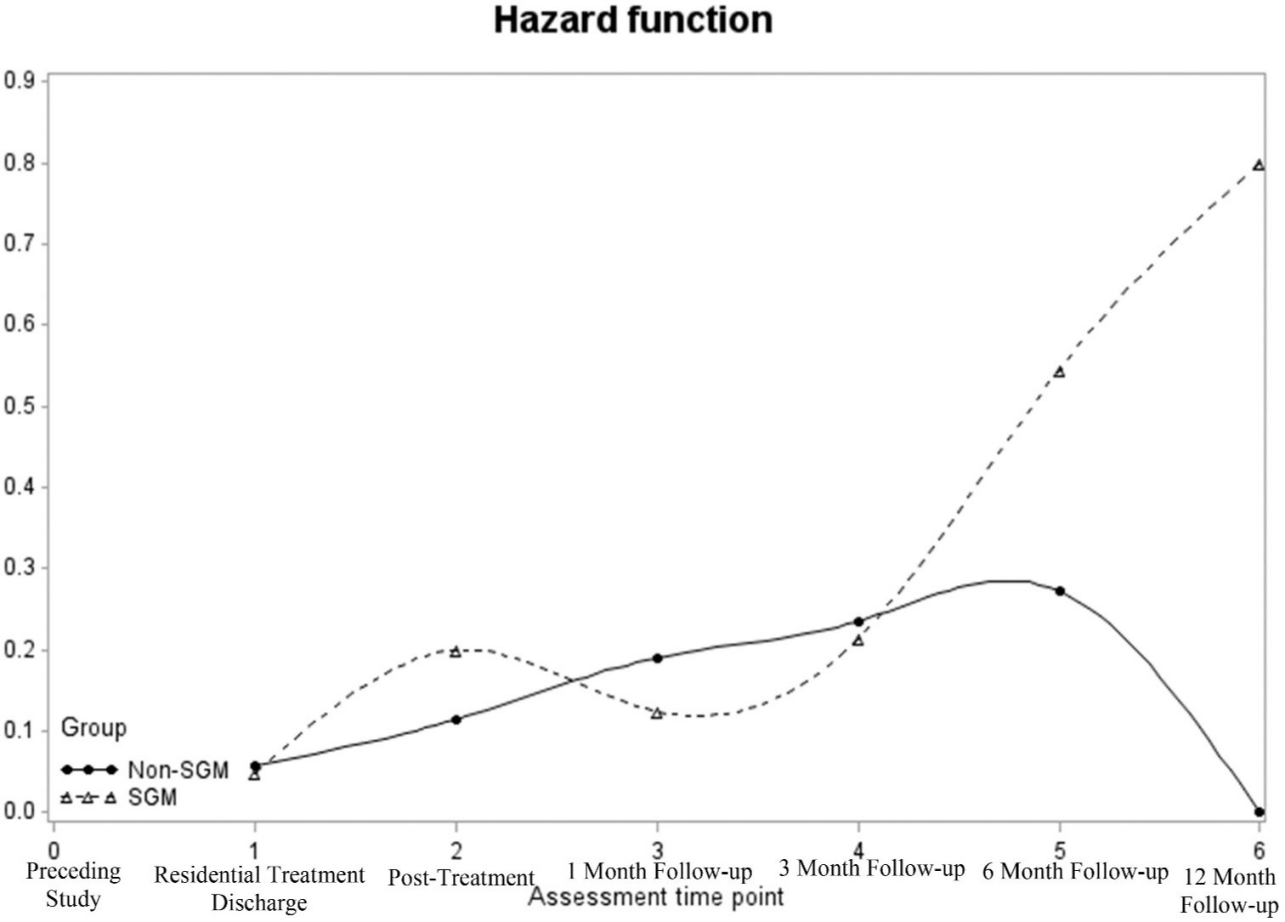
Hazard function (i.e., risk of substance use) by SGM status from residential discharge to 12-month follow-up.

Four participants were missing data at all six follow-up time points and were excluded from the survival analysis, resulting in a sample size of *n* = 56. At the end of 12-month follow-up, the survival rate (i.e., the percentage of the sample who were at risk of reusing but did not reuse substances) was 20.5% across the entire sample (Figure 1). In the non-SGM group, the survival rate was 37.6% compared with 4.8% in the SGM group. The hazard function shows that the risk of reusing substances was similar between the two groups through the 3-month follow-up, after which point the groups diverged (Figure 2).

To formally evaluate group differences, a survival analysis with discrete time indicated that the overall predictive model was significant (*χ*^2^ = 25.56, *p* = .001). The individual coefficients indicated that SGM individuals had a non-significantly higher hazard of substance reuse than non-SGM individuals over the course of the study period (log odds = .54, *p* = .11; Table 2). This indicates that SGM individuals had a 71.8% increase in the hazard of reusing substances during the study period. Similarly, the log odds estimates of reuse across subsequent time points (as compared to the reference of residential discharge) were highest at 1-month follow-up (log odds = 1.24, hazard ratio = 244.6, *p* = .09) and lowest at the 12-month follow-up (log odds = .25, hazard ratio = 27.8, *p* = .83).

### Frequency of SU

A count model with a random intercept and slope was used to predict the frequency of SU. In the model without covariates, SGM status was a significant predictor of the intercept (log odds = 3.62, *p* =.02), indicating that SU occurred more frequently in the SGM group than the non-SGM group at baseline. Neither the main effect of time (log odds = .03, *p* = .20) nor the time × SGM status interaction was significant (log odds = −.02, *p* = .60), suggesting a non-significant increase in the frequency of SU over the follow-up period that did not differ between groups. The model adjusting for baseline covariates produced similar findings (see Table 3).

### Problems associated with SU

**Figure 3.**
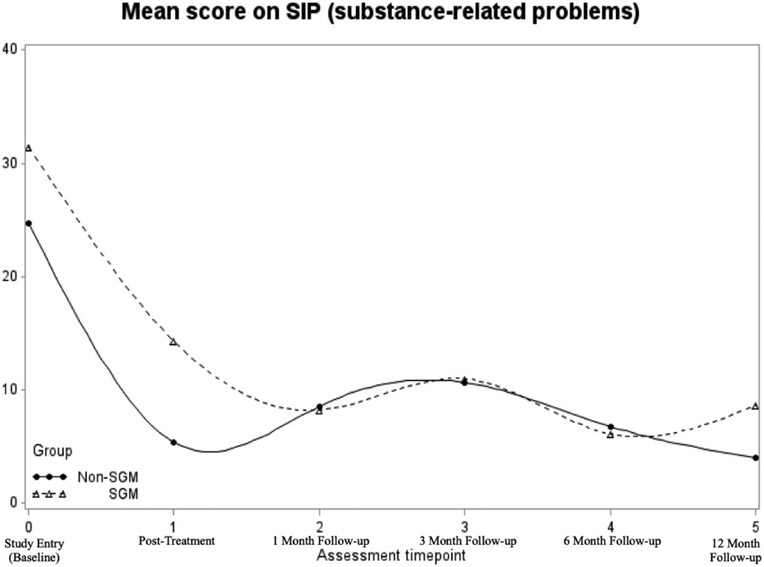
Mean score on substance related problems using the SIP by SGM status from study entry to 12-months follow-up.

A quadratic trajectory with a random intercept and slope provided the best fit for the data. There was a significant linear (*B* = −.81, *p* < .001) and quadratic (*B* = .008, *p* = .005) effect of time. The effect of SGM status was not a significant predictor of the intercept or any of the time effects (all *p*s > .10) in models with and without covariates (see Table 4). This indicates that on average, individuals in both groups experienced fewer SU-related problems over time, but the rate at which the problems decreased slowed over time (see Figure 3).

## Discussion

This study examined the role of SGM status on SU outcomes among a multiply minoritized population of people with HIV attending a combined inpatient-outpatient treatment program. We found a survival rate of 20.5% regarding substance reuse for the entire sample and a statistically non-significant, but potentially clinically relevant difference in survival rates between the non-SGM (37.6%) versus SGM (4.8%) groups. Further, the frequency of SU remained stable across the follow-up period for the entire sample, with both non-SGM and SGM groups experiencing significant reductions in problems related to SU.

Our finding that just over 20% of the entire sample at risk did not reuse substances parallels prior research (Mooney et al., 2014). However, our statistical approach using survival analysis provides a more accurate conceptualization of reuse than prior studies that have used a simple percentage of the sample that relapsed (Greenwood et al., 2001; Mooney et al., 2014). While we did not find any significant differences between SGM and non-SGM groups on any SU outcome, the survival rate of 4.8% in the SGM group, compared with 37.6% in the non-SGM group, could have important clinical implications if replicated in future research.

This finding is consistent with other studies that have found worse treatment outcomes among SGM individuals, including lower rates of treatment completion and abstinence after treatment (Senreich, 2007, 2009, 2011). The hazard function shows that through the 3-month follow-up, the SGM and non-SGM groups show similar risk. However, the risk of reuse diverged between the two groups at the 6- and 12-month follow-ups. It may be that certain risk factors for SU, such as having a social network that uses substances, may be more prominent among SGM individuals. Or, ongoing and cumulative SGM-related discrimination, which is commonly experienced by this population (Rice et al., 2021), may lead to reusing substances after several months.

This study has numerous strengths including its focus on a hard-to-reach, multiply minoritized sample in need of SU intervention efforts as well as the inclusion of an objective measure of SU (urinalysis). This study also used a longitudinal, prospective design for a mixed inpatient-outpatient treatment program with a 12-month follow-up and included several SU treatment outcomes. This study is limited by missing data at follow-ups due to a difficult-to-retain population and an overall small sample size. The small sample size required combining sexual and gender minorities into one group. Although prior research has taken this approach (Antin et al., 2018; Stojanovski et al., 2018) sexual and gender minorities experience different psychosocial stressors (Bockting et al., 2013; Clements et al., 2006; Puckett et al., 2023). Future work should capture their unique experiences in SU treatment.

Moreover, the study did not measure stigma or discrimination, both of which are highly relevant to a multiply minoritized population and their experience of health inequalities (Hatzenbueher et al., 2013). Finally, while study interventionists, who were clinical psychology doctoral candidates, had training in cultural competence, this is not necessarily part of treatment as usual in SU facilities. Future research should evaluate a variety of SU treatment outcomes for SGM individuals that are delivered by standard treatment providers.

Overall, the goal of this study was to examine how SGM status impacts SU outcomes in a multiply minoritized sample after a combined inpatient-outpatient treatment program using a robust study design. While results indicated no statistically significant differences between SGM and non-SGM individuals, we observed a potentially clinically meaningful difference in the survival rate of substance reuse (i.e., of those at risk of reusing, the percent who abstained from use). SGM individuals may demonstrate a lower abstinence rate at the 12-month follow-up compared to non-SGM individuals. Future work must replicate this finding and explore interventions specific to multiply minoritized SGM populations.

## Figures and Tables

**Table 1.** Baseline demographic and clinical characteristics for the overall sample and by SGM status.

Characteristic	Total sample (*N* = 60)	SGM *(n* = 21)	Non-SGM (*n* = 39)	*p*
Age, *M (SD)*	45.38 (7.81)	42.38 (8.32)	47.00 (7.15)	.02
% Female (n)^¶^	53.3 (32)	52.4 (11)	53.9 (21)	.91
% African American (n)	96.7 (58)	90.5 (19)	100 (39)	.11
% Employed (n)	91.7 (55)	85.7 (18)	94.9 (37)	.33
% Married or cohabitating (n)	10.0 (6)	9.5 (2)	10.3 (4)	1.00
Years since HIV diagnosis, *M (SD)*	1.67 (7.68)^a^	12.36 (6.82)^b^	9.66 (8.10)^c^	.24
CD4 count, *M (SD)*	420 (254)^d^	422 (243)^e^	419 (263)^f^	.97
Prior residential treatment episodes, *M (SD)*	3.43 (3.11)^d^	4.70 (3.71)^e^	2.68 (2.45)^f^	.01
SIP-AD, *M (SD)*	27.19 (13.07)^g^	31.48 (12.47)	24.69 (12.92)^h^	.06

*Note*. ^¶^Sex assigned at birth (male, female, other). ^a^*n* = 38. ^b^*n* = 18. ^c^*n* = 30. ^d^*n* = 54. ^e^*n* = 20. ^f^*n* = 34. ^g^*n* = 57. ^h^*n* = 36. SIP-AD = Short Inventory of Problems – Alcohol and Drugs; SGM = sexual and gender minorities.

**Table 2.** Discrete time survival analysis from residential discharge to 12-months follow-up.

Parameter	Log odds estimate [95% CI]	*SE*	*p* value	Hazard ratio^¶^
Intercept	−3.26 [−4.51, −2.01]	.64	<.001	–
SGM group	.54 [−.13, 1.21]	.34	.11	71.8
Residential discharge (reference) Post-treatment	−.90 [−.55, 2.35]	.74	.22	145.5
1-month follow-up	1.24 [−.17, 2.65]	.72	.08	244.6
3-month follow-up	1.21 [−.34, 2.76]	.79	.12	235.5
6-month follow-up	1.17 [−.67, 3.01]	.94	.21	221.9
12-month follow-up	.25 [−1.97, 2.46]	1.13	.82	27.8
Assessment period length	.01 [−.007, .03]	.01	.24	1.04
Assessment period length squared	<.001 [−.00005, .00003]	<.001	.71	–

*Note*. ^¶^Interpreted as the percent increase in the risk of reusing substances relative to the comparator. SGM = sexual and gender minorities.

**Table 3.** Log odds model of TLFB frequency over time from residential discharge to 12-months follow-up.

	SGM only model	Final multivariable model
Predictor	*Log odds (SE)*	*Log odds (SE)*
Intercept	−7.94 (1.23)***	−7.25 (1.21)***
Time (per week)	.03 (.03)	.03 (.03)
SGM	3.62 (1.53)*	2.46 (1.63)
Age		.12 (.08)
Prior residential treatment		.42 (.22)^†^
Time × SGM	−.02 (.03)	.001 (.04)
Time × Age		−.002 (.002)
Time × Prior residential treatment		−.009 (.005)^†^

*Note*, ^†^*p* < .10. *p < .05. **p < .01. ***p < .001. TLFB = Timeline Follow Back; SGM = sexual and gender minorities.

**Table 4.** Quadratic model of the SIP total (substance-related problems) over time from baseline (study intake) to 12-month follow-up.

	SGM only model	Final multivariable model
Predictor	*B (SE)*	*B (SE)*
Intercept	22.64 (2.16)***	23.60 (2.34)***
Time (per week)	−.81 (.18)***	−.76 (.20)***
SGM	5.76 (3.48)	3.37 (4.09)
Age		.07 (.24)
Prior residential treatment		1.36 (.62)*
Time × SGM	−.24 (.26)	−.24 (.31)
Time × Age		−.003 (.02)
Time × Prior residential treatment		−.05 (.05)
Time × Time (quadratic effect)	.008 (.003)**	.007 (.003)*
Time × Time × SGM	.003 (.004)	.003 (.005)
Time x Time x Age		< −.001 (<.001)
Time × Time × Prior residential treatment		.001 (.001)

*Note*. ^†^*p* < .10. *p < .05. **p < .01. ***p < .001. *B* = unstandardized beta; SGM = sexual and gender minorities.

## Footnotes

1.* This paper uses the term sexual and gender minority (SGM) due to its common use in this discipline’s body of literature. However, the authors recognize the recent social justice movement away from this term in favor of LGBTQ+ or sexual and gender diverse, especially amongst the population in question.

2.* The term “homosexual” was used in the study at the time of data collection for participants’ self-identification as gay or lesbian, though this term is no longer viewed as the most appropriate/commonly used term. We refer to these identities as gay or lesbian.

3.* We did not control for baseline differences between groups as we did in the trajectory analyses because participants with missing values were excluded from the analyses, resulting in a smaller sample size. A sensitivity analysis with the baseline differences added as covariates did not substantively change the results.

4.* Formula is 100(exp(estimate)-1).
